# Data on fluoride contamination in potable water in alluvial plains of district Panipat, Haryana, India

**DOI:** 10.1016/j.dib.2018.09.031

**Published:** 2018-09-18

**Authors:** Lakhvinder Kaur, Madhuri S. Rishi

**Affiliations:** Department of Environment Studies, Panjab University, Chandigarh, India

**Keywords:** Fluoride, Groundwater, Alluvial aquifer, Fluorosis

## Abstract

This data set reveals the fluoride concentration level of an alluvial aquifer of Panipat district Haryana India. The whole district of Panipat relies on groundwater for its agricultural, industrial and domestic purposes. Fluoride concentration in the study area varied from 0.5 mg/L to 5.95 mg/L with an average of 1.6 mg/L. 42.9% of the groundwater samples have shown higher fluoride concentration in groundwater than the permissible level prescribed by World Health Organisation and Bureau of Indian Standards. The spatial distribution map of fluoride has interestingly shown contrast between western and eastern parts of the region. Higher fluoride concentration (1.00–5.95 mg/L) in groundwater is witnessed in western half whereas the eastern half had comparatively lower concentration of fluoride ranging from 0.5 mg/L to 3.0 mg/L with maximum area having concentrations up to 1.5 mg/L. Major part 52.23% of Panipat district has shown high fluoride concentration in groundwater than the permissible level. It is further suggested that prolonged intake of groundwater with fluoride concentration higher than the permissible levels may cause dental or skeletal fluorosis in the locals.

## Specification table

**Table:** 

Subject area	Environmental Earth Sciences
More specific subject area	Hydrogeo-chemistry
Type of data	Table and Figure
How data was acquired	The groundwater samples were collected after 10–15 min pumping in pre washed high quality HDPE bottles. The pH, EC and TDS were recorded on site. Calcium, Magnesium, Sodium, Potassium, Carbonate, Bicarbonate, Sulphate, Chloride and Fluoride were analysed in the laboratory.
Data format	Analysed
Experimental factors	Fluoride content in groundwater samples was determined according to the method described in Standard Methods for the Examination of Water and Wastewater 21st edition
Experimental features	Fluoride concentration in groundwater samples was assessed by sodium 2-(parasulphophenylazo)-1,8-dihydroxy-3,6-naphthalene disulphonate (SPADNS) method using spectrophotometer.
Data source location	Panipat District, Haryana, India
	Geographical Coordinates: 29°10’15”: 29°30’25” North to 76°38’30”: 77°09’15” East
Data accessibility	Data available with this article
Related research articles	The fluoride content up to 0.5 mg/L is essential human supplement for stronger teeth and bone whereas beyond 1.5 mg/L causes acute to chronic skeletal and dental fluorosis [1], [2], [3], [4], [5]. Worldwide 200 million people have fluoride linked health problems due to consumption of high fluoride in groundwater [6]. Considering health effects linked with intake of fluoride rich water several techniques such as precipitation, electro-dialysis, ion exchange, reverse ion exchange and adsorption for fluoride removal in water have been evaluated in various studies [7], [8], [9], [10]. Adsorption method for fluoride removal is considered simplest and cost effective [10], [11], [12], [13]. In India, the incidences of skeletal and dental fluorosis has increased manifold due to high fluoride content in drinking water [14]. Occurrence and behaviour of fluoride in igneous rock terrain is well established [15] whereas its presence in alluvial aquifers is not well known [16], [17].

## Value of the data

• The spatial distribution of the data delineates the groundwater vulnerability zones with respect to fluoride linking it to the health hazards.
• The data set will help in understanding the correlation of fluoride with other major ions and cations and thus inferring the source of its origin.
• The fluoride data set will not only be useful for the environmental researchers and scientists but will be of great help to the water related policy makers and administrators to execute various groundwater related works.

## 1 Data

**Fig. 1:**
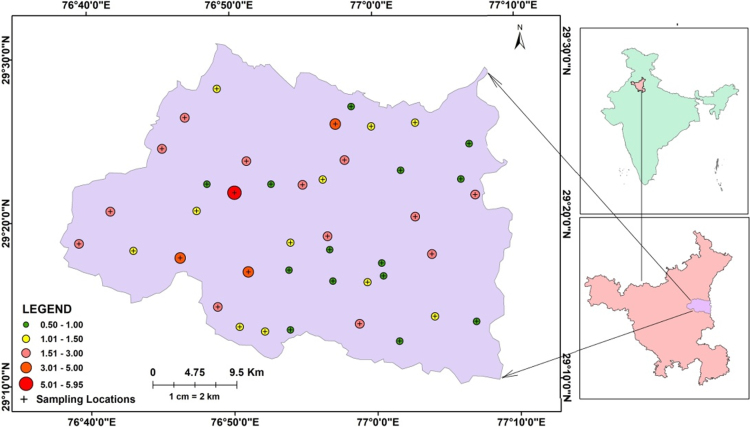
Location Map along with fluoride concentrations in Panipat District, Haryana.

**Fig. 2:**
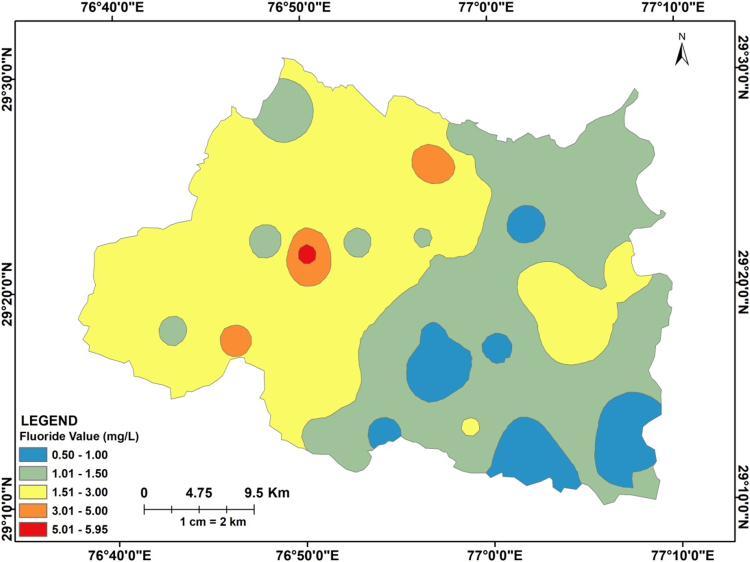
Map showing spatial distribution of fluoride in the analysed groundwater of Panipat district Haryana, India (post-monsoon 2015).

**Fig. 3:**
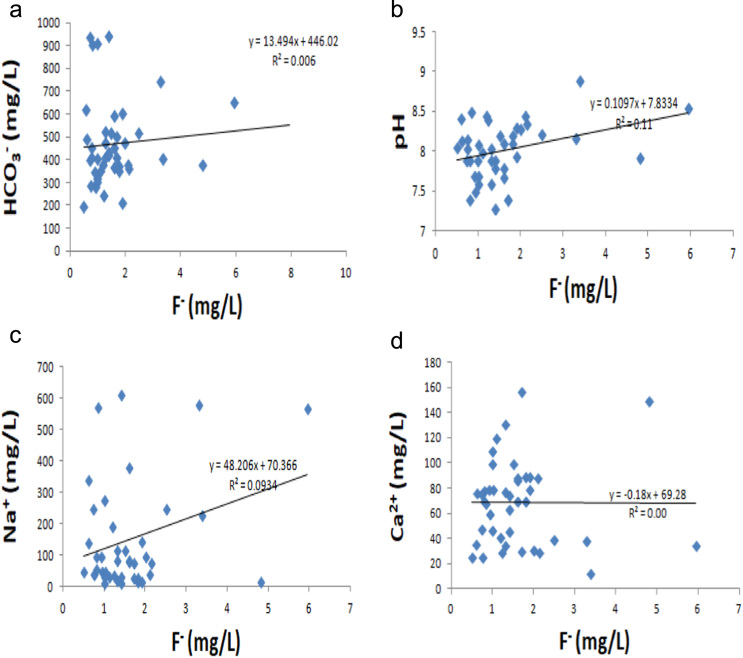
(a)–(d) representing inter-ionic relationships between F^−^ versus ${\mathrm{HCO}}_{3}^{-}$, F^−^ versus pH, F^−^ versus Na^+^ and F^−^ versus Ca^2+^ respectively.

Fluoride content (mg/L) in the groundwater samples of Panipat district, Haryana, India is expressed in the Fig. 1. Summary statistics of several groundwater quality parameters and the percentage of samples above prescribed limits are given in Table 1. Fig. 2 shows the spatial distribution of fluoride content in the study area. Table 2 exhibits the percent area under various categories of fluoride concentration. Fig. 3(a)–(d) exhibits the inter-ionic relationships of fluoride with $HCO_{3}^{-}$ , pH, Na^+^ and Ca^2+^ respectively.

**Table 1:** Summary statistics of the groundwater quality parameters used in the present data set.

**Parameter**	**Min**	**Max**	**Mean**	**Permissible limit (WHO & BIS)**	**No. of samples above permissible limit**	**% of samples above permissible limit**
pH	7.29	8.89	8.0	6.5–8.5	2	4.8%
TDS	260	2160	691.0	2000	1	2.3%
F^−^	0.5	5.95	1.60	1.5	18	42.9%
${\mathrm{HCO}}_{3}^{-}$	195	940	467.6	600	8	19.0%
Ca^2+^	13	157	69	200	Nil	Nil
Na^+^	15	613	147.3	200	10	23.8%

Total No. of samples = 42

**Table 2:** Percent area under various categories of fluoride concentration in Panipat district, Haryana, India.

**Categories**	**Min value (mg/L)**	**Max value (mg/L)**	**Area (km**^**2**^**)**	**% Area**
	**Range (mg/L)**			
1	0.5	1.00	109	8.64
2	1.01	1.50	494	39.13
3	1.50	3.00	630	49.90
4	3.01	5.00	28	2.17
5	5.00	5.95	2	0.16
Total			1263	100

## 2 Experimental design and methodology

### 2.1 Sample collection and analytical procedures

Panipat district is located between 29°10’15”: 29°30’25” North and 76°38’30”: 77°09’15” East, in Haryana, India. The area under investigation has almost homogenous geological nature and is completely covered by old and new alluvium deposits of quaternary to recent age, consisting of clay and sand [18]. Total 42 groundwater samples were collected in the pre-washed bottles after 10–15 min pumping, from the study area during post-monsoon 2015. The pH, TDS, EC was analysed on the spot by using hand held potable Hanna (HI 98194) multi-parameter instrument. The samples were filtered using Whatman filter paper No. 42 before storing in the sampling bottles. For cation analysis the groundwater samples were acidified using concentrated HNO_3_ to pH 2. The analysis of the calcium (Ca^2+^), magnesium (Mg^2+^), sodium (Na^+^), potassium (K^+^), bicarbonate (${\mathrm{HCO}}_{3}^{-}$), chloride (Cl^−^), sulphate (${\mathrm{SO}}_{4}^{2-}$) and fluoride (F^−^) in the laboratory was carried out using [19] methods. The F^−^ in the groundwater samples was analysed using sodium 2-(parasulphophenylazo)-1,8-dihydroxy-3,6- naphthalene disulphonate (SPADNS). The Na^+^ and K^+^ in the groundwater samples was analysed using Flame photometer. The (Ca^2+^), (Mg^2+^) and (${\mathrm{HCO}}_{3}^{-}$) in the groundwater samples was analysed using titration methods. The results of the analysis were further examined for the cation-anion balance and the cation-anion balance of the groundwater quality parameters was within 0–5%. The groundwater sampling locations and F^−^ concentration map and F^−^ spatial variability map were prepared by using Arc GIS 10.4.1.

The spatial distribution map of fluoride in groundwater was prepared by employing inverse distance weighting (IDW) interpolation as it was the best performer than the other interpolation methods having least mean error (ME) value of 0.015 and root mean square error (RMSE) of 1.183. Further, the percent area under various fluoride concentration values was calculated on the basis of IDW method is given in Table 2. The inter-ionic relationship graphs for F^−^ versus ${\mathrm{HCO}}_{3}^{-}$ , F^−^ versus pH, F^−^ versus Na^2+^ and F^−^ versus Ca^2+^ were plotted by using Excel 2007. The inter-ionic relationships of F^−^ with ${\mathrm{HCO}}_{3}^{-}$, pH, Na^+^ and Ca^2+^ are shown in Fig. 3(a)–(d) respectively.

### 2.2 Inter-ionic relationship plots for F^−^ versus ${\mathrm{HCO}}_{3}^{-}$, F^−^ versus pH, F^−^ versus Na^+^ and F^−^ versus Ca^2+^

See Fig. 3.
